# A Mixed L2 Norm Regularized HRF Estimation Method for Rapid Event-Related fMRI Experiments

**DOI:** 10.1155/2013/643129

**Published:** 2013-05-12

**Authors:** Yu Lei, Li Tong, Bin Yan

**Affiliations:** China National Digital Switching System Engineering and Technological Research Center, Zhengzhou 450002, China

## Abstract

Brain state decoding or “mind reading” via multivoxel pattern analysis (MVPA) has become a popular focus of functional magnetic resonance imaging (fMRI) studies. In brain decoding, stimulus presentation rate is increased as fast as possible to collect many training samples and obtain an effective and reliable classifier or computational model. However, for extremely rapid event-related experiments, the blood-oxygen-level-dependent (BOLD) signals evoked by adjacent trials are heavily overlapped in the time domain. Thus, identifying trial-specific BOLD responses is difficult. In addition, voxel-specific hemodynamic response function (HRF), which is useful in MVPA, should be used in estimation to decrease the loss of weak information across voxels and obtain fine-grained spatial information. Regularization methods have been widely used to increase the efficiency of HRF estimates. In this study, we propose a regularization framework called mixed L2 norm regularization. This framework involves Tikhonov regularization and an additional L2 norm regularization term to calculate reliable HRF estimates. This technique improves the accuracy of HRF estimates and significantly increases the classification accuracy of the brain decoding task when applied to a rapid event-related four-category object classification experiment. At last, some essential issues such as the impact of low-frequency fluctuation (LFF) and the influence of smoothing are discussed for rapid event-related experiments.

## 1. Introduction

In the last decade, multivoxel pattern analysis (MVPA) has become a widely used analysis method in cognitive neuroscience especially in decoding brain activities at different states [1–4]. MVPA mainly focuses on single-trial blood-oxygen-level-dependent (BOLD) responses to identify different brain states. In some experiments, to obtain an effective and reliable classifier or computational model, numerous samples should be collected using rapid event-related designs [3]. However, for rapid event-related designs, the overlapping of BOLD signals in the time domain encumbers the extraction of a real trial-specific BOLD response, which is important for MVPA. Hence, the accurate estimation of a trial-specific BOLD response is a challenging problem in rapid event-related MVPA.

Traditional estimating approaches are mainly classified into two groups. Model-based methods involve prior hemodynamic response function (HRF), whereas model-free methods have no assumptions on the shape of HRF. Model-based methods differ in the assumptions of the shape of HRF, such as the canonical double gamma function [5], Poisson function [6], radial basis function [7], and inverse logit function [8]. Previous reports revealed the capability of HRFs in the traditional univariate statistical analysis especially in activation-based analysis. However, most brain state decoding experiments or information-based analysis aim to obtain fine-grained spatial activation patterns that can help improve the performance of our decoding model [9]. Therefore, an accurate estimation that reflects real neural activities is necessary to obtain more fine-grained spatial activation patterns. In these cases, we cannot ignore the high variation in the temporal responses of different voxels across individuals as well as across tasks, regions of the brain, and different days within individuals [10]. Hence, model-free methods that are more sensitive and accurate have been widely used [11, 12].

For a model-free method, a voxel-specific HRF contains one free parameter for each time point. Thus, an HRF of arbitrary shape of each voxel that provides much more flexibility in data analysis can be obtained. In a model-free method, the first step is always to estimate a voxel-specific HRF and use this HRF to deconvolve BOLD signals [13]. When estimating a voxel-specific HRF, the BOLD response is often assumed to be a linear time-invariant (LTI) system [14]. Then, one of the main solutions is to represent the HRF with a linear combination of basis functions [15, 16]. Another solution is to treat the HRF at each point as a free parameter [17]. This paper alternatively focuses on the latter one. Modeling low-frequency fluctuation (LFF) is another problem in HRF estimation that should be addressed [18]. The linear drift in the obtained images is a challenging problem in fMRI data analysis because of the poor HRF estimates. A simple strategy for removing linear drift is to detrend time-series data as a preprocessing step [19, 20]. Alternatively, LFF may be modeled in a nuisance matrix consisting of some basis functions as regressors. This strategy enables not only linear detrending but also LFF removal to some extent [21], resulting in a more flexible and efficient detrending model.

Given that BOLD images have high noise, regularization is a popular technique that allows constraints to be imposed on HRF estimates to suppress the impact of noises when employing a parameter-free model. The smooth finite-impulse response (FIR) method [22] is a good example of regularization to smooth estimates. Tikhonov regularization may also be used to impose smoothness [23]. In [24, 25], Tikhonov regularization is combined with generalized cross-validation (GCV) to reduce the computational burden involved in parameter selection. Accordingly, Tikhonov regularization is also used in this paper. However, smoothness is only one of the local features of a signal, which could not reflect the global structure of a signal. Therefore, in extremely rapid event-related experiments, considering only smoothness is not enough to suppress the overlapping of different events, resulting in deformed HRF shape. Hence, we add an additional L2 regularization component into the estimation model with Tikhonov regularization, called mixed L2 norm (MN) regularization. Using this regularization method, we cannot only retain the smooth feature of HRFs but also prevent the significant overlapping of adjacent events. Furthermore, this method is a parameter-free model, indicating that it is adaptive to the variability of HRFs across voxels and individuals.

We first outlined the HRF and response estimation methods, especially the proposed MN estimation method and the classification approach used to assess their performances. All methods were applied to four-category object classification data to compare the classification accuracy. We also compared the classification performances between object responsive (OR) voxels and voxels in the early visual cortex. Finally, we discussed the role of LFF and the impact of smoothness in MVPA.

## 2. Materials and Methods

### 2.1. Subjects

Ten healthy subjects (six males and four females) participated in this fMRI study. The study was approved by the Institutional Review Board of China National Digital Switching System Engineering and Technology Research Center. All subjects provided written informed consent and had normal vision.

### 2.2. Stimuli

The stimuli consisted of four categories (car, animal, building, and human face) of color images, including 50 different images in each category. All images were cropped to the center (700 pixels × 700 pixels) and placed onto a gray-scale background.

Visual stimuli were rear-projected onto a screen in the scanner bore using a luminance-calibrated LCD projector driven by a PC. The subjects viewed the screen from a mirror. The display resolution was 1024 × 768, and the stimulus presentation script was written using MATLAB (The Mathworks) and Psychtoolbox 3.0 (http://psychtoolbox.org/).

### 2.3. Experimental Design

Each subject participated in three task runs, four localizer runs, and one retinotopic mapping run. In the task runs, images were presented in a 4 s stimulus trial. In each trial, an image was first presented for 2 s, and the gray background was presented for the last 2 s. Each presentation consisted of an image being periodically flashed ON-OFF, where ON corresponds to the presentation of the image for 200 ms and OFF corresponds to the presentation of the gray background for 200 ms. The first two task runs consisted of 70 distinct images randomly presented once for each time. The last task run consisted of 60 distinct images also randomly presented once for each time. After every five stimulus trials, a blank trial that lasted for 4 s was conducted as a break.

In localizer runs, the subjects were presented with blocks of images for each category. Each run consisted of 12 blocks, with 6 task blocks and 6 control blocks. The task block lasted the same time as the control block for 30 s. Each localizer run consisted of six images randomly selected from the same image category. Each task block consisted of an image being periodically flashed ON-OFF, where ON corresponds to the presentation of the image for 200 ms and OFF corresponds to the presentation of the gray background for 200 ms. The OR voxels were a set of voxels that were strongly activated in at least one localizer run (*t*-test, *P* = 0.005, family-wise error corrected).

Another standard retinotopic mapping run with polar stimuli was performed to delineate the early visual areas on a flattened cortex.

### 2.4. Data Acquisition

The data were collected using a 3-T GE Discovery 750 (General Electric, Fairfield, CT, USA) scanner with a standard head coil at the Imaging Center of Henan Province. For each subject, a standard gradient-echo-planar imaging series was used to collect functional images with the following parameters: repetition time (TR), 2000 ms; echo time (TE), 30 ms; field of view, 220 mm × 220 mm; matrix size, 64 × 64; 39 slices; slice thickness, 3.5 mm; flip angle (FA), 80°; and voxel size, 3.4 mm × 3.4 mm × 3.5 mm. In addition, a high-resolution three-dimensional T1-weighted anatomical image was acquired (TR, 8.268 ms; TE, 3.24 ms; FA, 12°).

### 2.5. Data Preprocessing

All fMRI data were preprocessed with SPM8 (Statistical Parametric Mapping, http://www.fil.ion.ucl.ac.uk/spm/software/spm8/) and REST (http://www.restfmri.net/). The first 10 volumes of each run were discarded because of the instability of initial magnetic resonance imaging signal and adaptation of subjects to the circumstance. Then, slice timing was performed on all functional images. The images were realigned to the first image in the first run for motion correction. We used REST to remove the linear drift in each run.

For retinotopic mapping analysis, FreeSurfer (http://surfer.nmr.mgh.harvard.edu/) was used to reconstruct a T1-weighted anatomical image. Then, the realigned retinotopic mapping images were registered to the anatomical image to obtain the registration file. The following retinotopic analysis was consistent with [26].

### 2.6. HRF Estimation

#### 2.6.1. Basic Model

In our model, the BOLD signal is assumed to be an LTI system with respect to the stimulus. Then, the measured BOLD time series is modeled as the convolution of an input signal. The hemodynamic response function is as follows:

(1) $$\begin{array}{c} y\left(t\right)=h\left(t\right)*s\left(t\right)=\sum_{k=0}^{L-1}h\left(k\right)s\left(t-k\right), \end{array}$$

where *y*(*t*) represents the fMRI time series, *h*(*t*) represents the HRF, *s*(*t*) represents the stimulus vector, and *L* indicates the discrete time length of the HRF. This model can also be rewritten in matrix form:

(2) $$\begin{array}{c} y=Sh, \end{array}$$

where **y** is a column vector of length *N* (*N* being the number of time points of the fMRI time series), **S** is the stimulus convolution matrix with a dimension of *N* × *L*, and **h** is a column vector of length *L*. The stimulus convolution matrix consists of shifted versions of a binary sequence, where ones indicate event occurrences.

Considering LFF and other noises, additional nuisance parts should be added to the above model. In this case, a set of Legendre polynomials of degrees 0 through 3, which are pairwise orthogonal, is used as regressors to compensate for LFF [21]. An autoregressive stochastic process of order one is also added [27]. Upon the incorporation of the nuisance parts, the HRF estimating model can be written as follows:

(3) $$\begin{array}{c} y=Sh+Pb+\epsilon , \end{array}$$

where **P** represents the nuisance matrix of dimension *N* × *B* consisting of Legendre polynomials of degrees 0 through 3, **b** is a column nuisance parameter vector of length 4, and **ϵ** represents the stochastic noise.

#### 2.6.2. Least-Square Estimation with AR (1) (LSAR) Noise Model

The LSAR of the HRF estimation problem can be achieved through the following steps (details can be found in [28]):

1. perform the ordinary least squares (OLS) method on $=W{\left[\begin{array}{cc} h & b \end{array}\right]}^{T}+\epsilon$, where $W=\left[\begin{array}{cc} S & P \end{array}\right]$, to obtain **ϵ**;
2. use **ϵ** to create a transformation matrix **L** with autocorrelation coefficients. Then, transform the original regression model $y=W{\left[\begin{array}{cc} h & b \end{array}\right]}^{T}+\epsilon$ using **L** to $Ly=LW{\left[\begin{array}{cc} h & b \end{array}\right]}^{T}+L\epsilon$;
3. conduct an OLS regression on the transformed formulation $\tilde{y}=\tilde{W}{\left[\begin{array}{cc} h & b \end{array}\right]}^{T}+\tilde{\epsilon }$ to obtain the real $\hat{h}$.

### 2.7. MN Estimation

Regularization is a common scalarization method for solving problems such as in the above-mentioned basic model. The most common form of regularization is called Tikhonov regularization, which results in a convex optimization problem [29]:

(4) $$\begin{array}{c} \text{minimize}\;\;{||Ax-b||}^{2}+\delta {||x||}^{2}. \end{array}$$

For various values of *δ* > 0, this problem has the following analytical solution:

(5) $$\begin{array}{c} x={\left(A^{T}A+\delta I\right)}^{-1}A^{T}b. \end{array}$$

This optimization problem can be extended in several ways. One useful extension is to add a regularization term with the form of ||**D** **x**|| in place of ||**x**||. In many cases, the matrix **D** represents an approximate differentiation or second-order differentiation operator; so ||**D** **x**|| represents a measure of the variation or smoothness of **x**. In the HRF estimation, we assume that it is smooth; so the second-order differentiation operator **D** is used in the regularization term to achieve a smooth result:

(6) $$\begin{array}{c} \text{minimize}\;\;{||Ax-b||}_{2}+\delta {||Dx||}_{2}. \end{array}$$

Another problem in the HRF estimation of rapid event-related experiments is the overlapping of adjacent events. When the interstimulus interval (ISI) is extremely short (e.g., 2 s), overlapping encumbers the calculation of the hemodynamic response function because the BOLD responses evoked by different events could not be separated successfully. To address the instability of the estimate, we also assume that the BOLD responses evoked by pulsed stimuli quickly return to the baseline. In this study, we assume that the BOLD responses return to the baseline 10 s after pulsed stimuli. In addition, the HRF should start from zero. Based on these assumptions, we can use a regularization term to constrain the solution. In our study, we aim to suppress the impact of overlapping, retaining the profile of HRF. Thus, to depict the character of the hemodynamic response, we use a regularization term as follows:

(7) $$\begin{array}{c} \text{minimize}\;\;{||Ax-b||}_{2}+\gamma \sum_{i=1,i>10}^{}x{\left(i\right)}^{2}. \end{array}$$

This formulation can be written in matrix form as follows:

(8) $$\begin{array}{c} \text{minimize}\;\;{||Ax-b||}_{2}+\gamma {||Cx||}_{2}, \end{array}$$

where

(9) $$\begin{array}{c} C=\left[\begin{array}{ccccccc} 1 & & & & & & \\ & 0 & & & & & \\ & & \ddots & & 0 & & \\ & & & 0 & & & \\ & 0 & & & 1 & & \\ & & & & & \ddots & \\ & & & & & & 1 \end{array}\right]. \end{array}$$

Considering the above-mentioned regularization terms, the mixed L2 norm (MN) regularization could be written as

(10) $$\begin{array}{c} \text{minimize}\;\;{||Sh+Pb-y||}_{2}^{2}+\delta^{2}{||Dh||}_{2}^{2}+\gamma^{2}{||Ch||}_{2}^{2}. \end{array}$$

Then, the HRF estimator derived is

(11) $$\begin{array}{c} \hat{h}=\left(S^{T}JS+\delta^{2}D^{T}D+\gamma^{2}C^{T}C\right)S^{T}Jy, \end{array}$$

where **J** = (**I** − **P** **P** ^**T**^). *δ* and *γ* are trade-off parameters to adjust the weight of the different regularization terms.

Using this new regularization term, smoothness and prior information about the HRF shape can be added to the estimation process. Hence, the noise caused by short ISI is removed.

### 2.8. Simulation Study: Tikhonov Regularization versus MN Regularization

**Figure 1:**
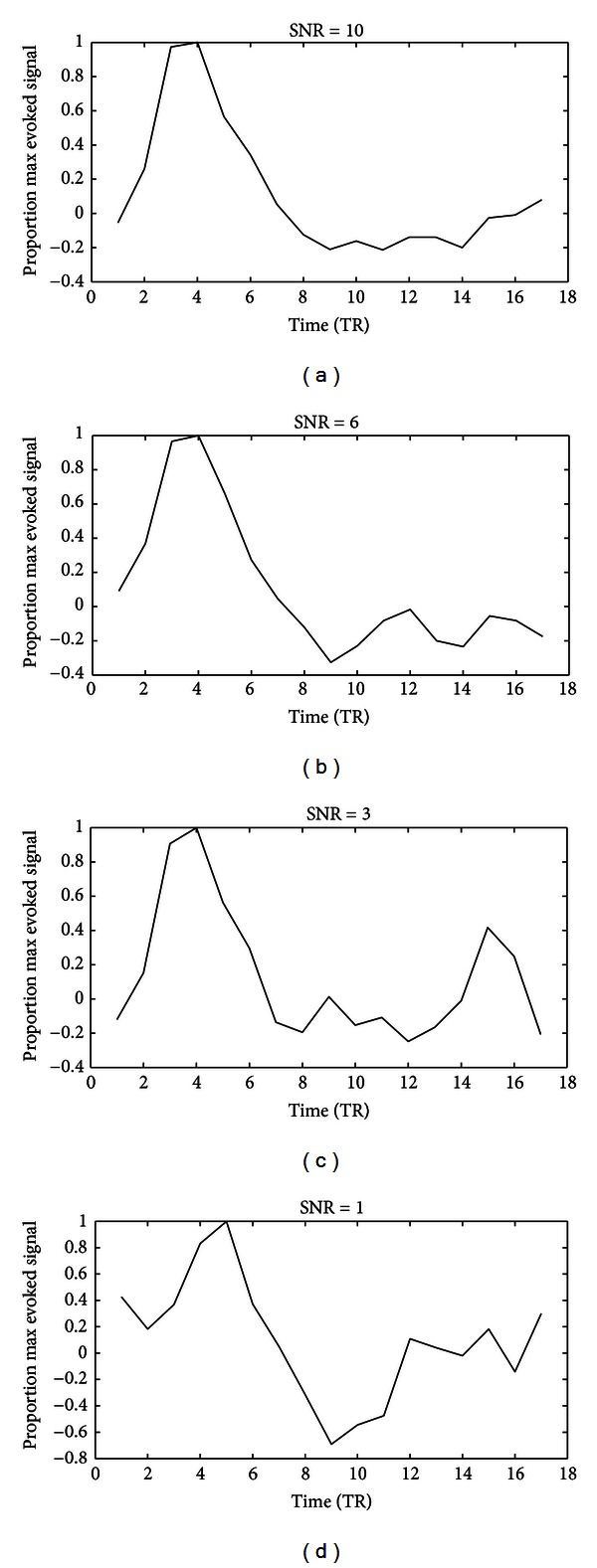
Simulation results of the HRF estimates using Tikhonov regularization. The *x*-axis indicates the time relative to the event onset (TR = 2 s), and the *y*-axis indicates the BOLD signal. Each HRF estimate was normalized by dividing it by its maximum value.

**Figure 2:**
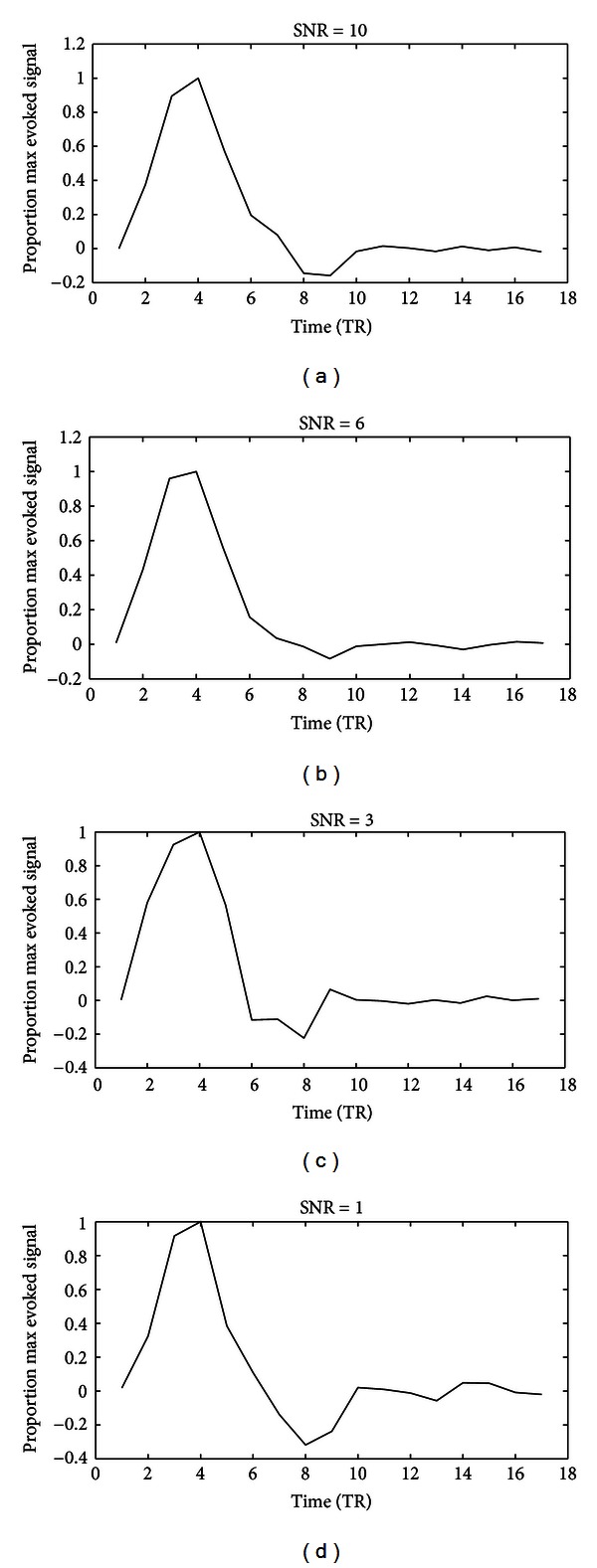
Simulation results of the HRF estimates using mixed-norm regularization. The *x*-axis indicates the time relative to the event onset (TR = 2 s), and the *y*-axis indicates the BOLD signal. Each HRF estimate was normalized by dividing it by its maximum value.

To understand the difference between Tikhonov regularization and MN regularization, we first compared the HRF estimation result in a simulation study.  Figure 1 shows the result of this simulation study using Tikhonov regularization, where the ISI was set to 2 s and the duration of stimuli was also set to 2 s. Time series was produced by convolving the stimulus vector with canonical double gamma HRF. Then, the Gaussian white noise of different signal-to-noise ratios (SNRs) was added to it. The result implied that with decreasing SNR, the overlapping increasingly destabilized the tail of the HRF estimates.  Figure 2 shows the result of the same simulation study using MN regularization. Compared with Figure 1, the result shows that when an additional regularization term was employed, most of the instability in the HRF estimates was suppressed. Based on this simulation study, the MN regularization method showed a great improvement in estimating HRF in a rapid event-related experiment.

### 2.9. Response Estimation Method

When voxel-specific HRFs are computed, we should deconvolve the time-series with the HRFs to obtain the real trial-specific BOLD responses. Reference [13] compared many deconcolving methods for multivoxel pattern classification analysis, such as FIR, ridge regression, partial least square, and support vector regression. In the following section, we will focus on the least square separate (LS-S) model.

**Figure 3:**
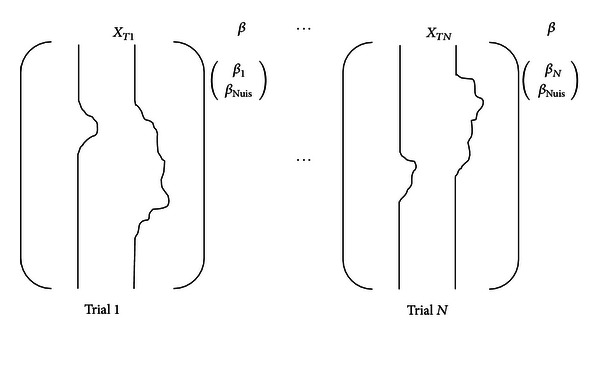
LS-S model. The design matrix has two regressors, one for the trial of interest and another for all other trials simultaneously. *X*_*T*1_ aims to obtain the activation estimate for trial 1. Therefore, a regressor is conducted for trial 1, and a second regressor is conducted for all other trials. The estimate for *β*_1_ based on this design is the estimate activation for trial 1. This method is repeated *N* times to obtain the estimates for all *N* trials.

In a rapid event-related fMRI data analysis, the traditional general linear model (GLM) suffers from collinearity induced by the correlation between trial-specific regressors. This collinearity could result in highly variable and unreliable estimates because of the lack of information that is unique to specific trials [13]. To reduce collinearity, we can modify the strategy of regressor construction or use regularization methods such as ridge regression [30] and partial least square [31]. In this paper, we use a regressor construction strategy called LS-S [13], which runs a GLM for each trial. The trial is modeled as the regressor of interest, and all other trials are combined into a single nuisance regressor. Thus, if we have *N* trials, we need to run the LS-S model *N* times to obtain each trial-specific response. The LS-S model is illustrated in Figure 3.

### 2.10. Classification Method and Statistical Analysis

As a widely used linear classifier, linear support vector machine (SVM) has been proved efficient in handling high-dimensional data. In our study, we also used the linear SVM based on LIBSVM [32] to compare the classification performances of the different estimation methods. The dataset was divided into five parts, and a fivefold “leave-one-out” cross-validation was applied to obtain the average classification accuracy. Lastly, the classification performances of the different methods were statistically compared using Wilcoxon signed-rank pair test.

## 3. Results and Discussion

This study aims to find an efficient method for estimating voxel-specific HRFs in a rapid event-related design fMRI study, which could deconvolve the BOLD time series to obtain the real BOLD activation signals associated with specific stimuli. The three task runs are divided into two parts. The third task run with 60 images is used to estimate voxel-specific HRFs and the other two task runs are used for classification analysis. In the following, we present the results of the analysis. Some essential aspects of this problem are also discussed.

### 3.1. Comparison of Different HRF Estimation Methods

To evaluate the performance of different HRF estimation methods in decoding brain states, we compared the classification accuracy of OLS, LS-AR (1), MN, and canonical double gamma HRF. Given the noise of the fMRI BOLD signals, we found that not all of the estimated HRFs of the voxels are acceptable. Considering this problem, we proposed a voxel selection criterion based on the prior knowledge about the BOLD responses. We assumed that *h*(0) was near zero and that the minimum value of normalized *h*(*t*) could not be less than −1. Furthermore, after 12 s, the BOLD responses should fall to the baseline. In other words, the voxel should be removed if it satisfies the following condition: |*h*(0)| > 0.3, max⁡⁡(*h*(*t* | *t* > 12 s)) > 0.4, and min⁡⁡(*h*(*t*)) > −1, where *h*(*t*) is the estimated HRF of a voxel.

Table 1 shows the mean number of voxels across all subjects before and after selection. The result indicates that good HRF estimates for all voxels could not be obtained in OR areas or early visual areas because of the noise in the time series. Therefore, invalid voxels were eliminated under the above-mentioned selection criterion. In addition, owing to the regularization, we obtained more voxels using the new MN estimation method.

**Figure 4:**
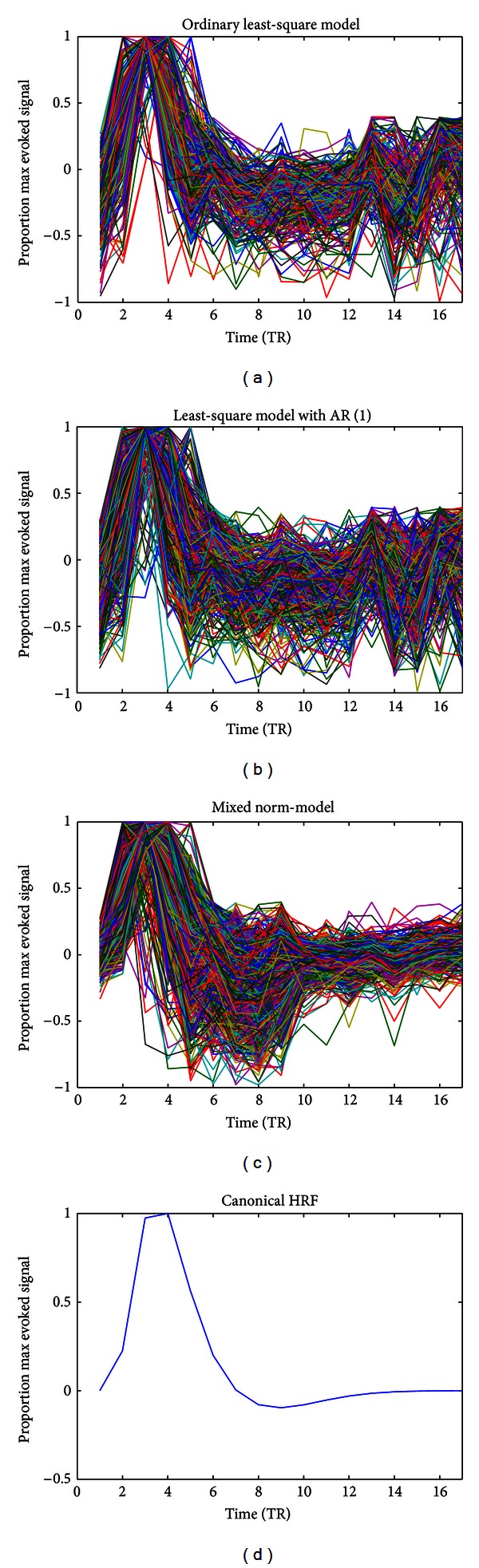
Inspection of the HRF estimates of the different methods. The lines in different colors represent the different HRF estimates of the voxels. The *x*-axis indicates the time relative to the event onset (TR = 2 s), and the *y*-axis indicates the BOLD signal. Each HRF estimate was normalized by dividing it by its maximum value.

In the simulation study, the MN method showed its capability in rapid event-related experiments. Here, the HRF estimation of the different methods was compared using real data.  Figure 4 shows one of the subjects' estimated HRFs in the OR areas. Figures 4(a) and 4(b) show the significant overlapping of the time series and a fake peak in the end of the estimated HRF using the OLS or LS-AR (1) method. However, in the MN estimation method, the fake peak was strongly suppressed because of the additional regularization term, as shown in Figure 4(c).  Figure 4(d) shows the canonical double gamma function [33].

**Figure 5:**
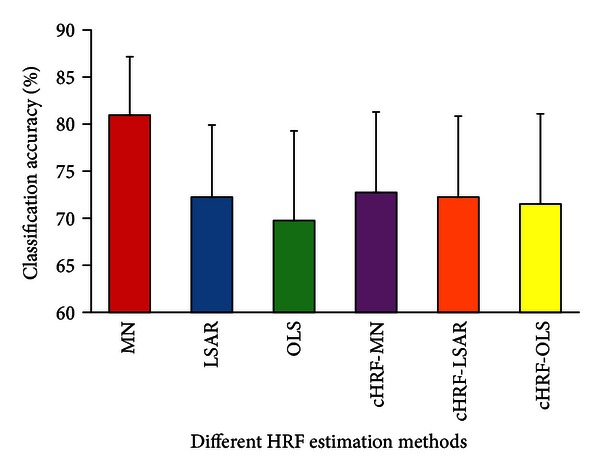
Bars show the classification accuracies of the different HRF estimation methods (cHRF-MN, canonical HRF with voxels selected by MN; cHRF-LSAR, canonical HRF with voxels selected by LSAR; and cHRF-OLS, canonical HRF with voxels selected by OLS). Error bars show the standard error of the mean classification accuracy across subjects.

The shape of the estimated HRFs intuitively showed the difference of the investigated methods. However, the shape could not be used to quantify this difference. Therefore, the classification accuracy based on real data was used to compare quantitatively the different methods.  Figure 5 shows the mean classification accuracy of the different HRF estimation methods across all subjects. For the different estimation methods, the classification results were 80.96%, 72.25%, 69.76%, 72.74%, 72.25%, and 71.51%. The results indicate that MN performed significantly better than the other five methods (Wilcoxon signed-rank pair test, *P* = 0.01).

**Figure 6:**
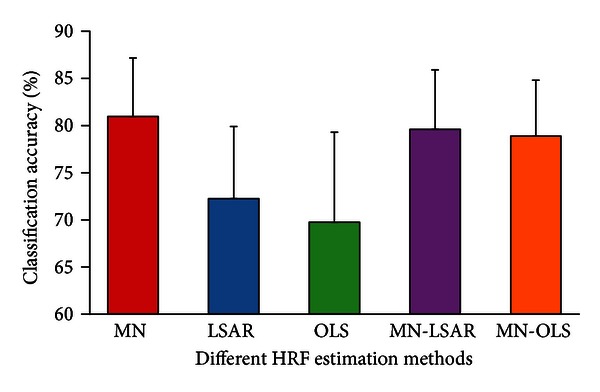
Effect of the size of voxel set. MN-LSAR, mixed-norm estimation was applied to the voxel set selected by LSAR; MN-OLS, mixed-norm estimation was applied to the voxel set selected by OLS. Error bars show the standard error of the mean classification accuracy across subjects.

The number of voxels after selection by different methods was different. The effect of the size of voxel set should therefore be considered. We applied MN to the voxel set selected by OLS or LSAR to investigate the impact of the size of voxel set.  Figure 6 shows that MN also performed significantly better than OLS and LSAR using the voxels selected by LSAR or OLS (Wilcoxon signed-rank pair test, *P* = 0.01). For the different estimation methods, the classification results were 80.96%, 72.25%, 69.76%, 79.59%, and 78.88%. This result indicates that the MN estimation method improved the classification accuracy and not the number of voxels.

Recent studies have illustrated the shape of HRF [5]. As a widely known model, the canonical HRF has been successfully used in fMRI studies, especially in univariate analysis or activation-based analysis. However, with the development of high-resolution fMRI, information-based analysis was applied for brain decoding [34]. In the present study, when voxel-specific HRFs were used, the classification accuracy significantly increased.

Many studies suggested that the fMRI time series has temporal autocorrelation between residual errors [27]. However, the present results indicated no significant differences in the classification accuracy between the least-square models with and without AR (1). 

In our MN estimation method, no assumption is made on the noise model, and the only task is the selection of regularization parameters. Considering that the selection of proper regularization parameters is one of the most important steps in solving regularization problems, many articles focused on the regularization parameter selection strategy to improve the performance of regularization, such as generalized cross-validation (GCV) [35], Bayesian information criterion (BIC) [36], and Akaike information criterion (AIC) [37]. In the present study, to simplify the problem, we selected the best one from a set of parameters. Although this strategy might not identify the best parameters, this method also performed significantly better than the other methods.

### 3.2. Impact of Smoothing on Brain Decoding

**Figure 7:**
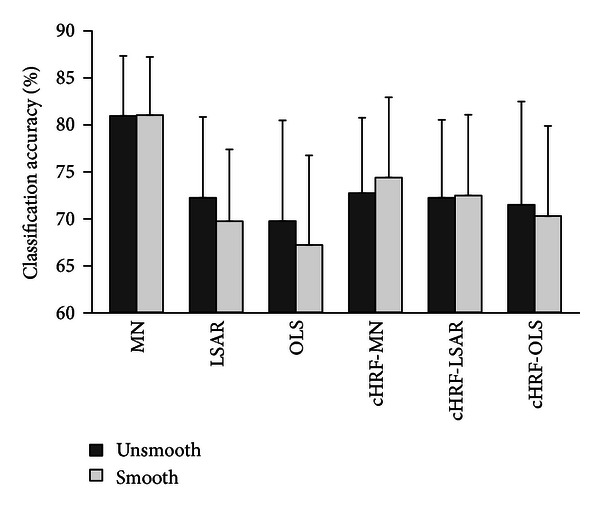
Comparison of smooth and unsmooth data. Classification accuracies estimated with fivefold leave-one-out cross-validation. Each method was applied to both unsmooth and smooth data to investigate the impact of smoothing. cHRF-MN, canonical HRF with voxels selected by MN; and cHRF-LSAR, canonical HRF with voxels selected by LSAR; cHRF-OLS, canonical HRF with voxels selected by OLS. Error bars show the standard error of the mean classification accuracy across subjects.

We compared the classification accuracy of the smoothed and unsmoothed data to investigate the impact of smoothing on brain decoding.  Figure 7 shows the classification accuracies of the different HRF estimation methods. For the smoothed data, the classification results were 81.03%, 69.74%, 67.23%, 74.39%, 72.49%, and 70.3%. For the unsmoothed data, the classification results were 80.96%, 72.25%, 69.76%, 72.74%, 72.25%, and 71.51%. The results implied no significant difference between the smoothed and unsmoothed data in brain decoding (Wilcoxon signed-rank pair test, *P* = 0.01). This conclusion is consistent with the finding of a previous study [38]. 

Smoothing is a standard preprocessing step in traditional activation-based or univariate analysis. However, in MVPA, whether smoothing should be conducted is unclear [38–40]. Many studies smooth the fMRI data before analysis to increase the SNR [41–43]. However, considering that smoothing may blur data, some studies omitted smoothing in analysis [1–3]. To preserve fine-grained pattern information, [39] suggested that smoothing should be omitted or strongly reduced.

Our result in this study implies that smoothing may not decrease the sensitivity and performance of brain decoding. However, this result does not mean that smoothing does not blur the fine-grained weak spatial information across voxels. A detailed explanation is given by [40]. If a study does not focus on subvoxel information sources or fine-grained information, smoothing would not matter.

### 3.3. Effect of LFF

**Figure 8:**
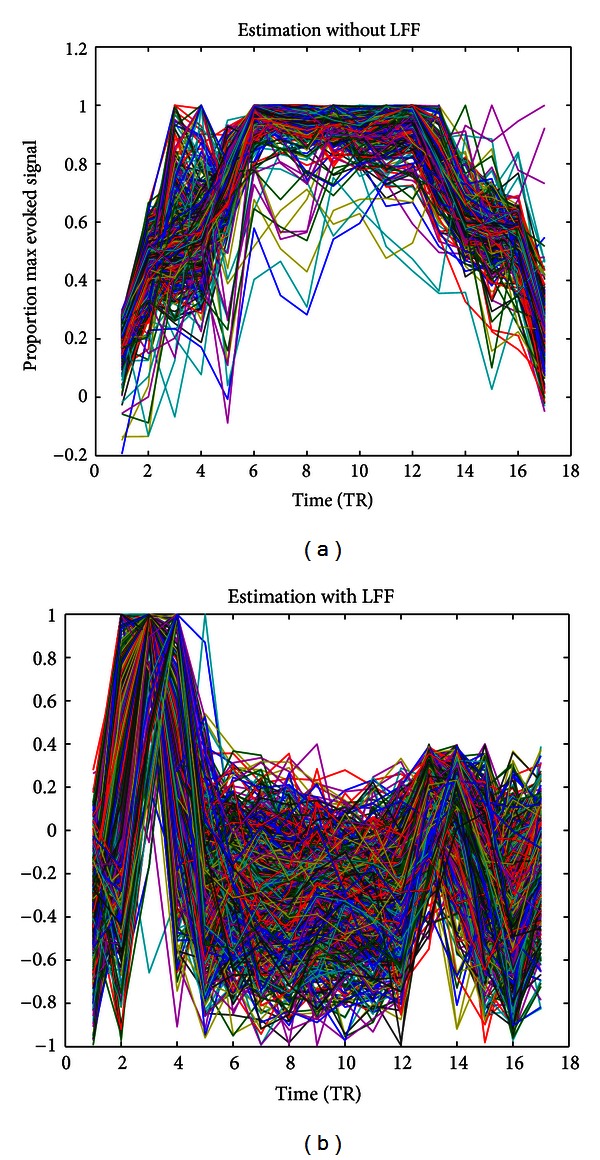
Comparison of HRF estimates with and without LFF. The lines in different colors represent the different HRF estimates of the voxels. The *x*-axis indicates the time relative to the event onset (TR = 2 s), and the *y*-axis indicates the BOLD signal. Each HRF estimate was normalized by dividing it by its maximum value.

The LFF compensation model is widely used in estimating voxel-specific HRFs.  Figure 8 shows a comparison of the estimated HRF using LS-AR (1) with and without the LFF model in one of our subjects. Based on the results, LFF had a large impact on HRF estimates. However, when we deconvolved the BOLD response from the fMRI time series, the impact of LFF was ignored. By contrast, in the MVPA analysis, wherein one of our goals is to exploit the information of weakly activated voxels, LFF significantly reduced the classification accuracy.

**Figure 9:**
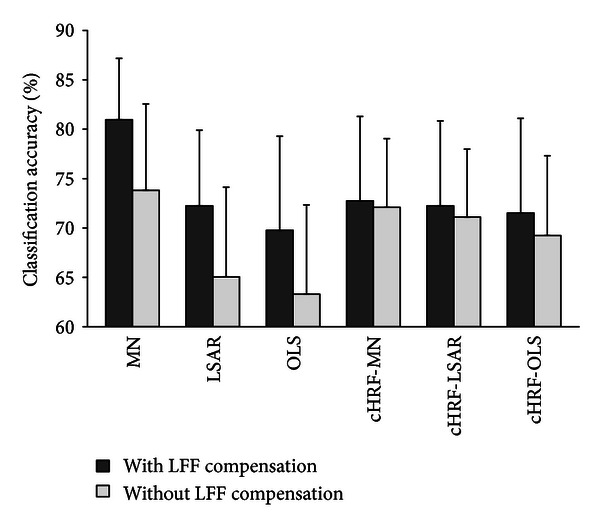
Comparison of classification accuracies with and without the LFF compensation component. Classification accuracies estimated with fivefold leave-one-out cross-validation. Each method was applied to both models with and without LFF data to investigate the impact of LFF. cHRF-MN, canonical HRF with voxels selected by MN; cHRF-LSAR, canonical HRF with voxels selected by LSAR; and cHRF-OLS, canonical HRF with voxels selected by OLS. Error bars show the standard error of the mean classification accuracy across subjects.

In the current study, we compared the classification performances of the response estimation method with and without the LFF compensation component.  Figure 9 illustrates the effect of LFF in brain decoding. For the model with LFF, the classification results were 80.96%, 72.25%, 69.76%, 72.74%, 72.25%, and 71.51%. For the model without LFF, the classification results were 73.81%, 65.04%, 63.3%, 72.09%, 71.1%, and 69.23%. These results indicated that the model with LFF compensation performed significantly better than that without LFF compensation (Wilcoxon signed-rank pair test, *P* = 0.01). Interestingly, no significant difference was found between the models with and without the LFF compensation component using canonical HRF. Our results implied that LFF plays an important role not only in HRF estimation but also in response estimation.

### 3.4. Classification Performances of Different Masks

**Figure 10:**
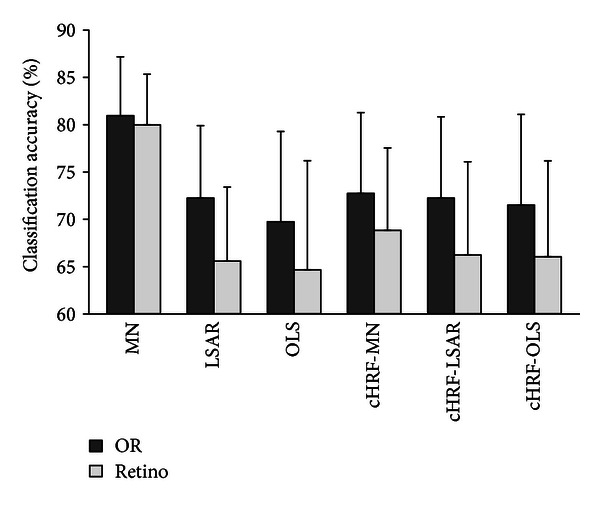
Comparison of classification accuracies in object responsive (OR) or early visual areas. OR voxels were strongly activated in localizer runs (*t*-test, *P* = 0.005, family-wise error corrected). Retinovoxels were located in the early visual area, which was delineated via retinotopic mapping analysis. Classification accuracies estimated with fivefold leave-one-out cross-validation. cHRF-MN, canonical HRF with voxels selected by MN; cHRF-LSAR, canonical HRF with voxels selected by LSAR; and cHRF-OLS, canonical HRF with voxels selected by OLS. Error bars show the standard error of the mean classification accuracy across subjects.

For each subject, we defined a mask of OR voxels in the occipital and temporal cortex that responded strongly in at least one of the four localizer runs. Then, nearly 3000 voxels were selected for each subject. The voxels in these areas were previously shown to provide information about object category [1, 44]. We also tested the classification accuracy in early visual areas, which were delineated by retinotopic mapping [26]. The classification accuracies in both masks are summarized in Figure 10. The classification results for the OR voxels were 80.96%, 72.25%, 69.76%, 72.74%, 72.25%, and 71.51%. The classification results for the retinovoxels were 79.99%, 65.59%, 64.67%, 68.83%, 66.24%, and 66.05%. The results showed that the OR voxels performed better than the retinovoxels in all six cases (significantly better in LSAR, OLS, cHRF-MN, cHRF-LSAR, and cHRF-OLS. Wilcoxon signed-rank pair test, *P* = 0.05).

These results demonstrated that the object category information has a distributed representation in the occipital and temporal areas. This information could improve the performance of classification. Therefore, localizer runs are necessary in object-related brain decoding experiments. Furthermore, in a brain-computer interface system with visual information, the spatially distributed information in large areas should not be ignored to obtain a better result.

## 4. Conclusions

In this paper, we propose a new HRF estimation method that uses Tikhonov regularization and additional shape regularization term to address the HRF estimation problem in rapid event-related experiments and suppress the overlapping of adjacent events. To test its performance, we applied this method to four-category object classification data. The results showed a significant improvement in classification performance, which proved that the new MN regularization HRF estimation method was more efficient than the others. Some essential issues in MVPA were also discussed in this paper, including the role of LFF in response estimation, the effect of data smoothing, and the differences in the classification accuracy between OR voxels and retinovoxels. Based on this work, we conclude that LFF compensation is necessary in MVPA analysis and that smoothing is an alternative. Moreover, spatially distributed information should be considered to obtain the best classification performance.

## Figures and Tables

**Table 1:** Mean number of voxels across all subjects used in different methods. Unselected voxels were all located in OR areas or early visual areas. Voxels were selected from the unselected voxel set based on the selection criterion. Object responsive (OR) voxels were strongly activated in localizer runs (*t*-test, *P* = 0.005, familywise error corrected). Retinovoxels were located in the early visual area, which was delineated via retinotopic mapping analysis.

	OLS	LS-AR (1)	MN
OR			
Unselected	2682	2682	2682
Selected	387 ± 127	425 ± 163	1334 ± 246
Retino			
Unselected	1290	1290	1290
Selected	221 ± 103	276 ± 112	721 ± 175
